# Tactile memory impairments in younger and older adults

**DOI:** 10.1038/s41598-024-62683-y

**Published:** 2024-05-23

**Authors:** Lilith-Sophie Lange, Anastasia Chrysidou, Peng Liu, Esther Kuehn

**Affiliations:** 1 Institute for Cognitive Neurology and Dementia Research (IKND), Medical Faculty, Otto-Von-Guericke University Magdeburg, Leipziger Straße 44, 39120 Magdeburg, Germany; 2 German Center for Neurodegenerative Diseases (DZNE), Otfried-Müller Straße 23, 72076 Tübingen, Germany; 3 Hertie Institute for Clinical Brain Research, Eberhard Karls University Tübingen, Otfried-Müller-Straße 27, 72076 Tübingen, Germany

**Keywords:** Long-term memory, Episodic memory, Consolidation, Hippocampus, Haptic, Psychology, Human behaviour

## Abstract

Human tactile memory allows us to remember and retrieve the multitude of somatosensory experiences we undergo in everyday life. An unsolved question is how tactile memory mechanisms change with increasing age. We here use the ability to remember fine-grained tactile patterns passively presented to the fingertip to investigate age-related changes in tactile memory performance. In experiment 1, we varied the degree of similarity between one learned and several new tactile patterns to test on age-related changes in the “uniqueness” of a stored tactile memory trace. In experiment 2, we varied the degree of stimulus completeness of both known and new tactile patterns to test on age-related changes in the weighting between known and novel tactile information. Results reveal that older adults show only weak impairments in both precision and bias of tactile memories, however, they show specific deficits in reaching peak performance > 85% in both experiments. In addition, both younger and older adults show a pattern completion bias for touch, indicating a higher weighting of known compared to new information. These results allow us to develop new models on how younger and older adults store and recall tactile experiences of the past, and how this influences their everyday behavior.

## Introduction

We are equipped with the astonishing ability to remember tactile experiences of the past, such as the surface of a tree bark or the kiss by our partner. This allows us to recognize common objects by touch^1^ and to memorize bodily experiences that guide our behavior^2^. Even though both tactile perceptual and episodic memory performance decreases with increasing age (for review^3^), the cognitive mechanisms of age-related changes in tactile memory are largely unexplored.

Age-related impairments in episodic memory performance are widespread and have been detected in visual memory^4^, spatial memory^5^, auditory memory^4^, and verbal memory^6^. Memory research has further evidenced age-related impairments in haptic exploration^7–11^, visuo-motor behavior^12^, and tactile-to-vision matching tasks^13^, but has rarely investigated tactile memory in isolation. This limits us in getting a detailed understanding of the cognitive mechanisms underlying the tactile component of age-related changes in object handling, body perception, and associated behaviors.

To understand the cognitive mechanisms that underlie age-related changes in tactile memory, we developed two novel behavioral paradigms (paradigm 1 introduced in experiment 1, paradigm 2 introduced in experiment 2) in which participants are asked to learn and recognize tactile patterns that are passively presented to their fingertip. Given the tactile patterns are presented passively (i.e., no active movement is required), and they are also not visible, both paradigms are suitable to investigate the cognitive mechanisms of tactile memory in isolation, and age-related changes thereof.

When designing the two experiments, we took into consideration that age-related declines in memory performance are often characterized by reduced precision and increased bias. Precision is a general property of memory systems and relates to the “uniqueness” of a stored memory trace that allows us to separate a given memory from all other occurrences^14^. Previous studies stress the presence of less precise memory representations in older adults. For example, it has been suggested that aging decreases the precision of visual memories^15^, spatial updating ^16^, and spatial recall ^17^. Conceptually, this has been related to the neural dedifferentiation hypothesis according to which reductions in the regional specificity and precision of neural representations relate to the reduced distinctiveness of memory encoding^18^. Also during tactile perception, older adults show larger tuning widths of tactile representations in contralateral primary somatosensory cortex (SI) in response to passive tactile stimulation^19^. However, the precision of tactile memories has not yet been compared behaviorally between younger and older adults. It is therefore unclear whether or not the “uniqueness” of a tactile memory trace reduces with age.

To investigate this, in experiment 1, we systematically varied the degree of similarity between one learned tactile pattern and several new tactile patterns that were passively presented to the fingertips of younger and older adults to identify the degree of dissimilarity that is needed by younger and older adults to successfully distinguish a stored tactile memory from a given tactile percept. This allowed us to test whether and to which extent the “uniqueness” of a tactile memory trace reduces with age.

With respect to bias, memory biases describe systematic shifts in memory recall patterns that influence the weighting of past and present experiences. Such biases can occur when the balanced interaction between two cognitive processes, pattern completion and pattern separation, is weighted towards one or the other^20–23^. More precisely, pattern completion mediates the retrieval of learned information based on partial cues of this information^24,25^. Pattern completion therefore allows, for example, recognizing a known building in the dark, when only partial visual information of this building is available. For the sense of touch, pattern completion may allow recognizing a tactile object when only partial tactile information of this object is provided, for example when the hand is cold or the object is wet. Pattern separation, on the other hand, mediates the reduction of interferences, that is, the representational overlap between two different inputs^23^. Pattern separation therefore supports, for example, the ability to distinguish a given building from a similar one viewed before by reducing interferences between similar memories. For the sense of touch, pattern separation may allow distinguishing the surface of a given object from a similar yet different one. Critically, pattern completion and pattern separation are two interacting cognitive processes, and the relative higher weighting of one of the other process causes memory biases: The higher weightening of pattern completion over pattern separation mediates a bias towards favoring the perception of known over novel experiences (due to the tendency to complete partial input to a stored memory trace rather than separating both), whereas the higher weightening of pattern separation over pattern completion mediates a bias towards favoring the perception of novel over known experiences (due to the tendency to separate novel input from stored memory traces rather than completing it). For the sense of vision, it has been shown that older adults show a bias towards pattern completion^22^. For the sense of touch, it is unclear whether or not a bias exists, and to what extent it changes with increasing age.

To investigate this, in experiment 2, we transferred a paradigm that has been used in vision research to the sense of touch. The visual paradigm has used pictures of rooms, such as a kitchen or or a library, to investigate the balance between pattern separation and pattern completion in younger and older adults^22^. In this study, either a full visual scene or a degraded version of known and new rooms, where only parts of the original room were visible, was shown. When asked to recognize the type of room in conditions of reduced visual information, younger adults equally often chose a known and a new room, except from the condition of lowest visual information, where they more often chose a new room. Older adults, on the other hand, had a tendency to more often choose a known room in conditions of low visual information, i.e., they showed a pattern completion bias^22^.

In order to adapt the paradigm by Vieweg et al.^22^ to the sense of touch, in experiment 2, we asked younger and older adults to recognize three learned tactile patterns among three learned and three new tactile patterns presented to the fingertip. Both learned and new tactile patterns were presented in either complete (full tactile information, all pins presented to the finger) or incomplete (reduced tactile information, pins missing) versions. This allowed us to investigate the ability of younger and older adults to complete a degraded version of a known tactile pattern to its full version (i.e., pattern completion), as well as the ability to separate a new tactile pattern (either complete or degraded) from all previously learned patterns (i.e., pattern separation) as well as the relative weighting between both processes. This allowed us to test whether tactile perception presents with a bias towards the past or the presence, or neither of the two, and to what extent this changes with increasing age.

To summarize, in experiment 1, we systematically varied the degree of similarity between one learned and several new tactile patterns to test on age-related changes in the “uniqueness” of a stored tactile memory trace. In experiment 2, we systematically varied the degree of stimulus completeness of both known and new tactile patterns to test on age-related changes in the weighting between known and novel tactile information. Both experiments provide critical new information on the question to what extent and under which experimental conditions older adults show alterations in tactile memory performance compared to younger adults. This allows us to develop new models on how older adults store and recall tactile experiences of the past, and how this influences their everyday behavior.

## Results

### Higher tactile detection thresholds in older adults

Before each of the two experiments started, we conducted a tactile detection task with both younger (18–30 years) and older (> 65 years) adults to later adjust the level of stimulus intensity at which the tactile patterns were presented to each individual person. In accordance with previous research^26^, analyses revealed that older adults have higher tactile detection thresholds compared to younger adults in both experiments with large effect sizes. In experiment 1, younger adults show a mean tactile detection threshold of 67.48% (SD = 7.4%), and older adults show a mean of 85.96% (SD = 17.22%). The difference between younger and older adults is statistically significant (*t*(19) = 3.82, *p* = 1.160*10^−3^, Cohen’s *d* = 1.39). In experiment 2, younger adults show a mean of 75.49% (SD = 11.63%), and older adults show a mean of 88.60% (SD = 17.97%). The difference is statistically significant (*t*(37) = 2.72, *p* = 9.850*10^−3^, Cohen’s *d* = 0.87). Note that 100% = 1.5 mm.

### The “uniqueness” of a tactile memory trace is only marginally lower in older compared to younger adults (experiment 1)

**Figure 1:**
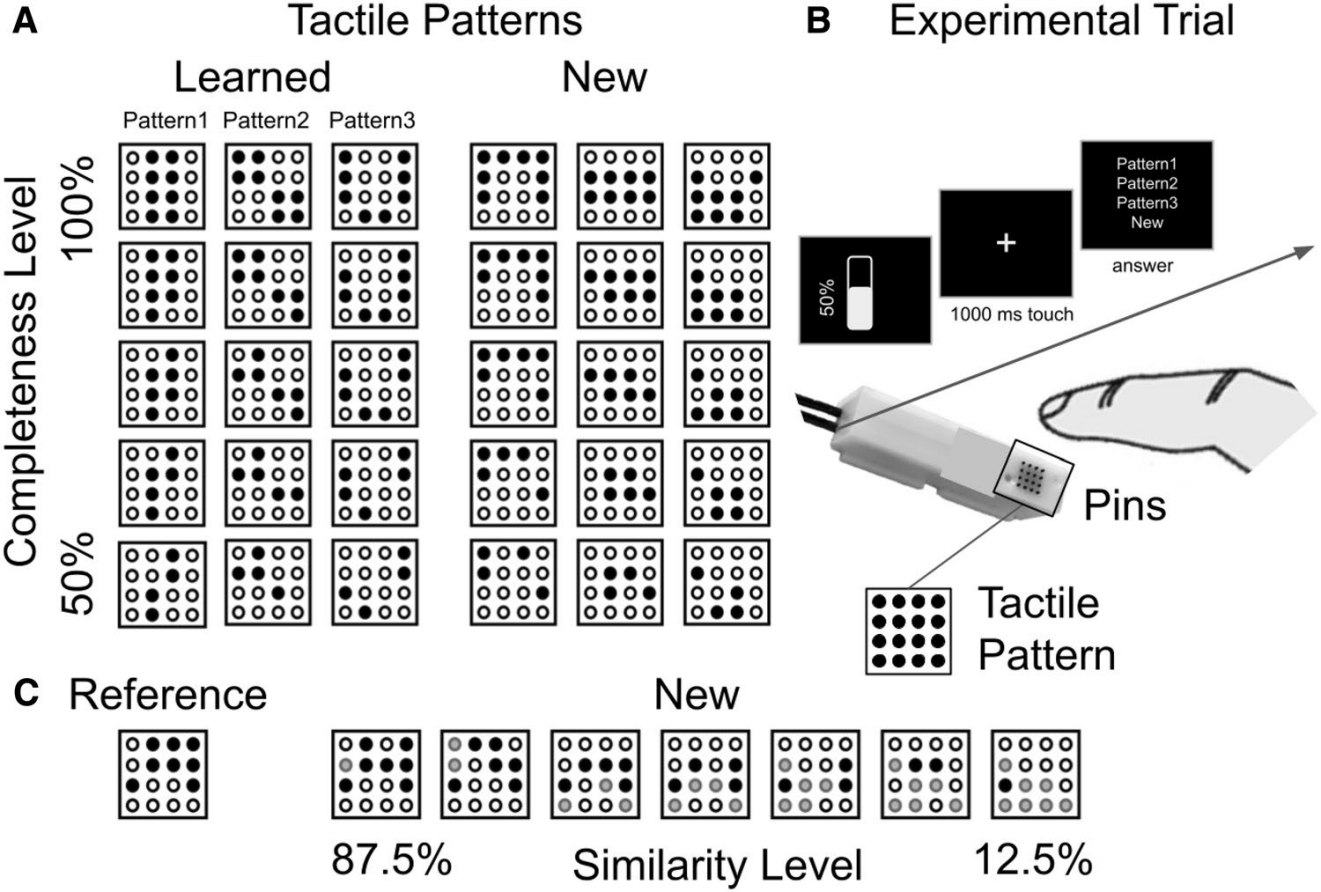
Stimuli and Experimental Setup. (**A**) Overview of experiment 2: The three ‘learned’ tactile patterns were learned during learning phases 1 and 2. The three ‘new’ tactile patterns were presented in the experimental phase and had to be dissociated from the three learned patterns. Black dots indicate the elevated and vibrating pins of each tactile pattern, white dots indicate the pins that were not elevated and did not vibrate. Both ‘learned’ and ‘new’ tactile patterns were either presented in full (completeness level 100%) or at reduced completeness (completeness levels 87.5%, 75%, 62.5%, 50%). (**B**) Experimental trial of experiment 2: Participants were informed of the completeness level of the tactile pattern via a bar on screen. After tactile stimulation, they were offered a 4-alternative-forced choice response (‘pattern1’, ‘pattern2’, ‘pattern3’, ‘new’). A Piezo Stimulator with a 4 × 4 stimulation module was used to create the tactile patterns. (**C**) Overview experiment 1: The ‘reference’ tactile pattern was learned during learning phase 1. The seven ‘new’ tactile patterns differed in 1–7 pins (i.e., in 87.5–12.5%) from the ‘reference pattern’. Black dots indicate elevated and vibrating pins that remained the same as in the ‘reference pattern’, white dots indicate the pins that were not elevated and did not vibrate, gray dots indicate pins that were elevated and vibrated, but differed from the ‘reference pattern’. The same stimulator as in experiment 2 was used (shown in (**B**)).

To test for age-related differences in the precision of tactile memories, in experiment 1, younger and older adults first learned to recognize one tactile pattern that was passively presented to their right index fingertip (‘reference pattern’, see Fig. 1C). After successful learning, either the ‘reference pattern’ or a new tactile pattern of varying levels of similarity to the ‘reference pattern’ (87.5%, 70%, 62.5%, 50%, 37.5%, 25%, 12.5% similarity, ‘new pattern’, Fig. 1C) was presented to their right index fingertip. Participants were asked to indicate whether the presented tactile pattern was ‘known’ or ‘new’ to them. The lower the level of similarity between the ‘new pattern’ and the ‘reference pattern’, the easier was the task. By systematically varying stimulus similarity between the ‘reference pattern’ and the ‘new pattern’, and by comparing performance between younger and older adults, we tested whether and to which extent the “uniqueness” of a tactile memory trace reduces with age.

Before testing these hypotheses, we conducted two control analyses to confirm that (i) participants perform above chance level in this task (note that this task is presented here for the first time), and (ii) participants perform better when tactile patterns are presented that are less similar to the reference pattern (proof-of-concept). Results confirm both assumptions and show that both younger and older adults perform above chance (chance level: 50%) for detecting the ‘reference pattern’ among the ‘new patterns’, with effect sizes exceeding the Cohen’s convention of a large effect^27^ (younger adults: M = 76.67%, SD = 12.34%, older adults: M = 81.33%, SD = 14.08%, see Supplemental Material Table 1 for effect sizes and confidence intervals (CI)). Also, as expected, a two-way ANOVA with the between factor age (younger, older) and the within factor condition (12.5%, 25%, 37.5%, 50%, 62.5%, 70%, 87.5%) on accuracies reveals a large effect size for the main effect of condition (F(3.03, 84.88) = 28.177, *p* = 8.17*10^−13^, η^2^ = 0.433), driven by higher accuracies for patterns less similar to the reference pattern compared to those more similar (see Supplemental Material Table 2 for detailed statistics).

**Figure 2:**
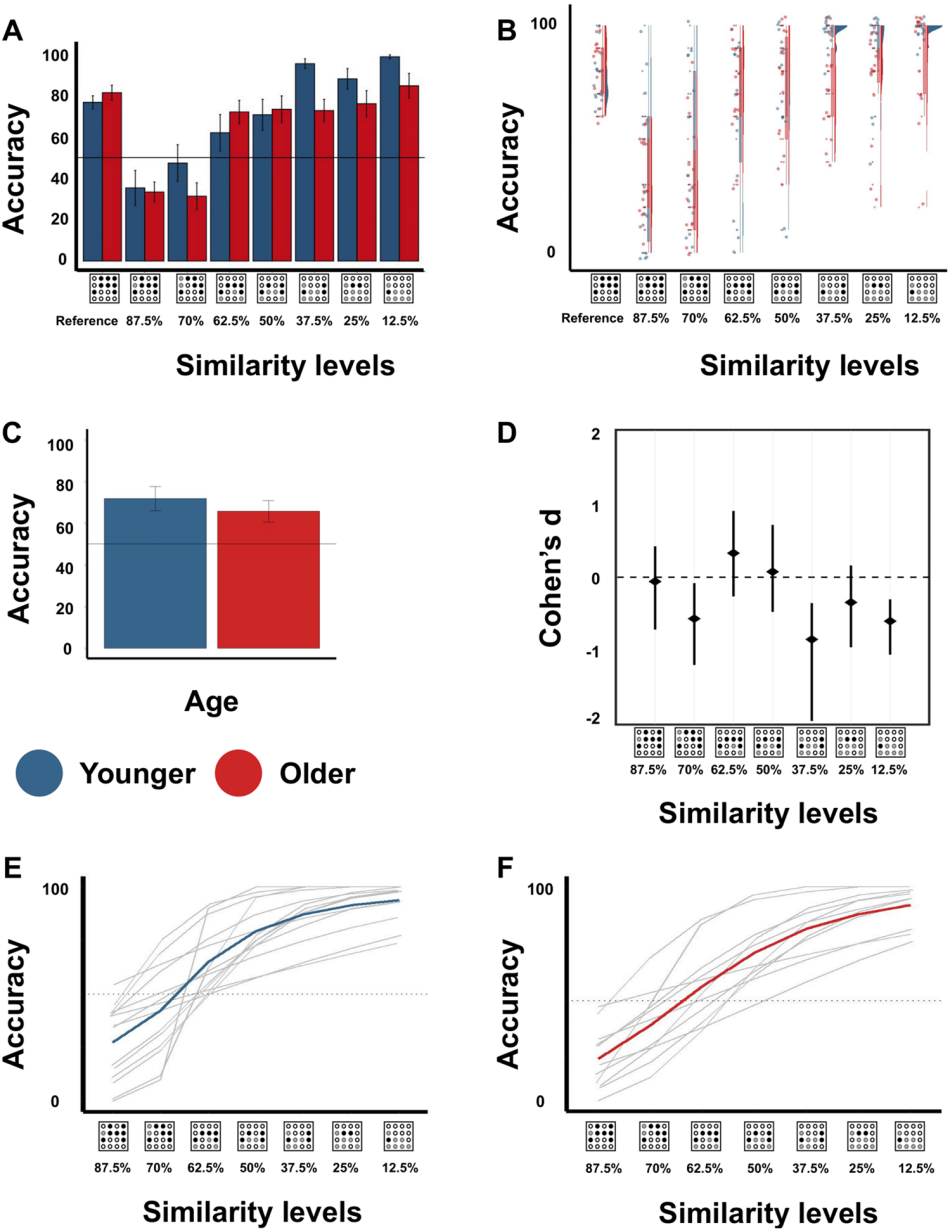
Precision to differentiate one learned tactile pattern from several new tactile patterns with varying similarity in younger and older adults (experiment 1). (**A**) Mean accuracy to correctly recognize the ‘reference pattern’ and the ‘new patterns’ plotted for the different similarity levels for younger (*blue*) and older (*red*) adults (mean ± CI). 50% marks chance level. (**B**) Raincloud plots for data shown in (**A**), individual data shown as colored dots: younger adults = *blue* and older adults = *red*. Boxplots are drawn within the interquartile range (box), medians are shown as vertical lines, whiskers connect the minimum and the maximum with the lower and the upper quartiles. (**C**) Main effect of age on accuracy to correctly recognize the ‘reference pattern’ and the ‘new pattern’ (mean ± CI). (**D**) Effect sizes (Cohen’s d) for data shown in (**B**) plotted for the different similarity levels. Negative values indicate lower performance of older compared to younger adults, positive values indicate better performance of older compared to younger adults. (**E**, **F**) Accuracy to correctly recognize a ‘new pattern’ after goodness-of-fit estimates for each individual of younger and older adults (*thin lines*), and mean curve for younger and older adults (*thick lines*, 50% thresholds: younger = 3.15, older = 3.42, *p* = .467, Cohen’s d = 0.36, *thin dotted lines* indicate 50% correct performance).

To investigate whether and to what extent the precision to recognize a learned tactile pattern is affected by age, we inspected the main effect of age and the interaction between age and condition of the above-introduced ANOVA. Results reveal that older adults show numerically lower accuracy in this task compared to younger adults (see Fig. 2A–C), however, this effect presents with a low effect size (F(1, 28) = 3.074, *p* = 0.091, η^2^ = 0.026, see Supplemental Material Table 1for effect sizes and CI). When inspecting effect sizes per similarity level, it is evident that older adults show specific impairments in the easier (lower similarity) conditions (see Fig. 2D). An exception is here the 70% condition where the effect size towards worse performance of older adults is also medium. Note that the interaction between age and condition presents with a low effect size (F(3.03, 84.88) = 1.901, *p* = 0.135, η^2^ = 0.049, see Fig. 2A,B). Other than expected, older adults therefore do not show specific impairments to recognize a new tactile pattern at high and middle similarity levels to the learned pattern, where it is most difficult to differentiate the ‘new patterns’ from the ‘reference pattern’, and where the threshold of “uniqueness” is defined. They also do not show a strong effect of generally worse performance. Rather, they perform poorer than younger adults particularly in the easier (lower similarity) conditions and therefore do not reach peak performance.

This was confirmed when estimating the 50% thresholds in younger and older adults at which more often ‘new’ was answered when in fact a new pattern was presented. Older adults show numerically lower precision than younger adults (i.e., higher 50% thresholds: younger: M = 3.15, older: M = 3.42, see Fig. 2E,F), but this difference presents with a low effect size (t(15) = 0.75, *p* = 0.467, Cohen’s *d* = 0.36). The extent to which the precision to recognize a learned tactile pattern is lower in older compared to younger adults is therefore low.

To clarify whether or not there is evidence in our data that the “uniqueness” of a tactile memory trace is reduced with age, we conducted a Bayes factor analysis to investigate if the likelihood for a difference in precision as estimated by the 50% threshold between younger and older adults is higher than the likelihood for no difference. Results show that it is 0.654 times more likely that there is a difference between younger and older adults than that there is no difference (error % = 0.003, 95% CI = [− 0.306, 1.118]). This indicates only anecdotal evidence for the assumption that the “uniqueness” of a tactile memory trace is lower in older compared to younger adults.

### Tactile memory is characterized by a pattern completion bias that is only marginally modulated by age (experiment 2)

To test whether tactile perception presents with a bias towards the past or the presence, or neither of the two, and to what extent this changes with increasing age, in experiment 2, a new cohort of younger and older adults was investigated, and a different paradigm was used. Participants here first learned to discriminate three tactile patterns that were passively presented to their fingertips from each other (‘learned patterns’, see Fig. 1A). In the experiment, the three ‘learned patterns’ and three new tactile patterns (that were not perceived before, ‘new patterns’) were presented either at full or at reduced tactile completeness levels (100%, 87.5%, 75%, 62.5%, 50%). Reducing tactile completeness was realized by removing pins from the patterns (100% completeness: 0 pin removed (8 pins left, complete pattern), 87.5% completeness: 1 pin removed (7 pins left), 75% completeness: 2 pins removed (6 pins left), 62.5% completeness: 3 pins removed (5 pins left), 50% completeness: 4 pins removed (4 pins left), see Fig. 1A). Participants were then asked to either identify the ‘learned pattern’, or to correctly recognize that the pattern was new (possible answers: ‘pattern 1’, ‘pattern 2’, ‘pattern 3’, ‘new’). This allowed us to compare the ability to correctly recognize known versus new tactile patterns under conditions of high or low stimulus completeness, and to test for the presence of memory biases and age-related changes thereof.

**Figure 3:**
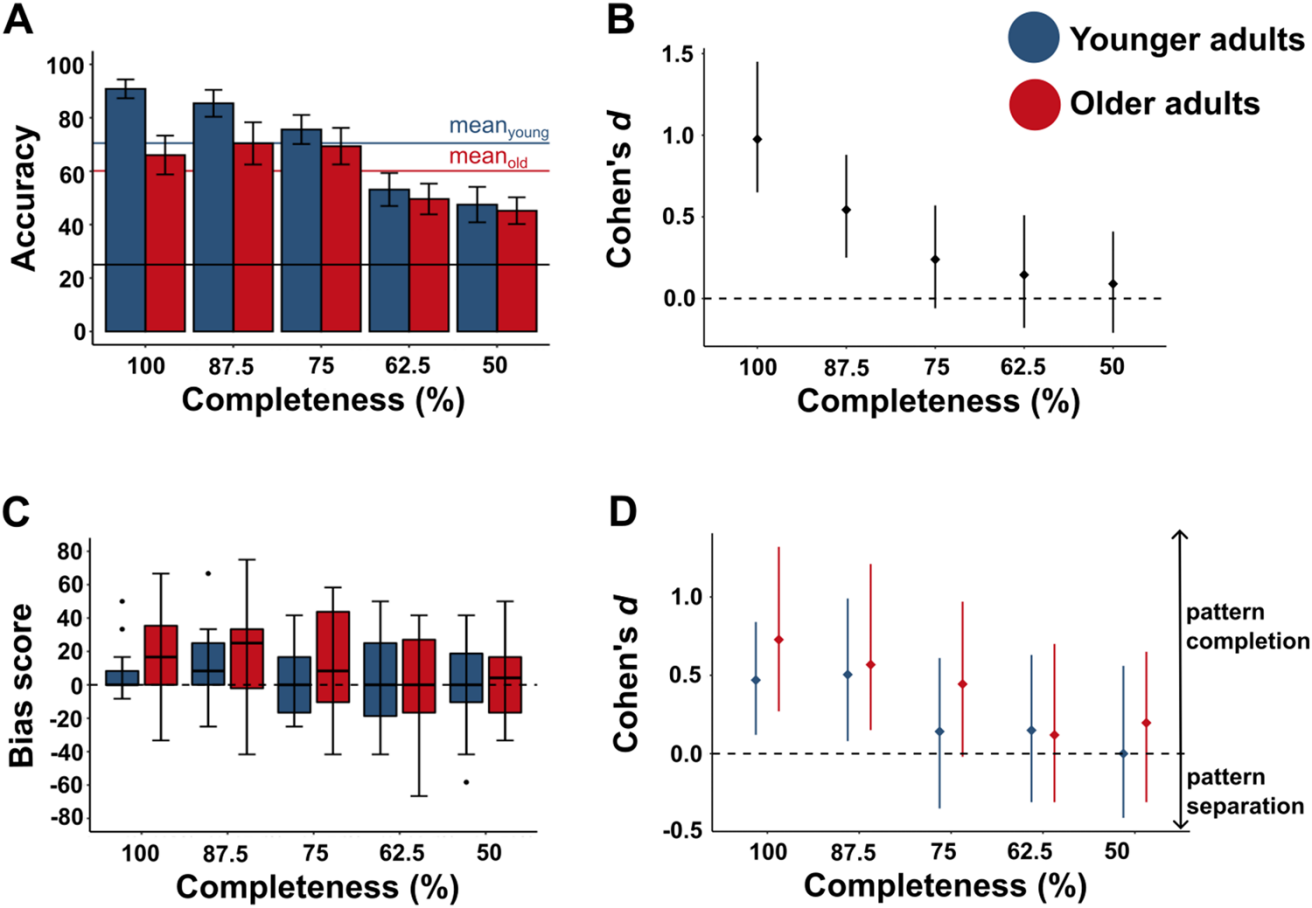
Performance to discriminate learned from new tactile patterns at different completeness levels in younger and older adults (experiment 2). (**A**) Mean accuracy to correctly recognize the learned patterns’ and ‘new patterns’ and the ‘new patterns’ plotted for the different completeness levels for younger (*blue*) and older (*red*) adults (mean ± CI). *Blue line* and *red line* indicate mean values for younger and older adults across completeness levels. Chance level is 25% (*black line*). (**B**) Effect sizes (cohen’s *d* ± CI) of data shown in (**A**). (**C**) Boxplots of bias scores for younger and older adults plotted for the different completeness levels. Bias scores were calculated by subtracting the accuracy in the new conditions from the accuracy in the learned conditions for each completeness level separately. (**D**) Effect sizes (cohen’s *d* ± CI) of the data shown in (**A**), compared to 0 (no bias). Positive values indicate a pattern completion bias, negative values indicate a pattern separation bias.

Before testing these hypotheses, we conducted two control analyses to confirm that (i) participants perform above chance level in this task (note that this task is presented here for the first time), and (ii) participants perform worse when stimulus completeness reduces (proof-of-concept). Results confirm both assumptions and show that both younger and older adults perform above chance (chance level: 25%) for detecting completely presented ‘learned patterns’ with large effect sizes (younger adults: M = 94.17%, SD = 5.48%, older adults: M = 75.42% SD = 16.10%, see details Supplemental Material Table 1 for effect sizes and CI). Also, as expected, the three-way ANOVA with the between-factor age (younger, older) and the within-factors condition (learned, new) and stimulus completeness (100%, 87.5%, 75%, 62.5%, 50%) on accuracies reveals a large effect size for the main effect of stimulus completeness driven by better performance for more complete patterns compared to less complete patterns (see Fig. 3A, see Table 1 for full ANOVA results and Table 2 for post-hoc comparisons).

**Table 1:** Results of three-way ANOVA on accuracy with the factors age, condition and completeness (experiment 2).

Effect on accuracy	F	*DFn*	*DFd*	*p*	ŋ^2^
Age	10.04	*1*	*38*	3.00*10^−3^*	0.07
Condition	7.44	*1*	*38*	0.01*	0.05
Completeness	107.46	*4*	*152*	2.84*10^−43^*	0.37
Age * Condition	1.21	*1*	*38*	0.279	0.01
Age * Completeness	10.27	*4*	*152*	2.18*10^−7^*	0.05
Condition * Completeness	3.19	*4*	*152*	0.02*	0.02
Age * Condition * Completeness	0.80	*4*	*152*	0.526	0.00

Shown are the main effects of age, condition and completeness on accuracy respectively, interaction effect between age and condition on accuracy, between age and completeness on accuracy, between condition and completeness on accuracy, and between age, condition and completeness on accuracy.

F = test statistic, *DF* = degree of freedom.

*p* = significance *p*-value, ŋ^2^ = effect size.

Significance is indicated by *.

**Table 2:** Results of post-hoc comparisons on accuracy between completeness levels (experiment 2).

Comparison		t	*df*	*p* (corr.)	Cohen’s *d*	CI	
						Lower	Upper
100%	87.5%	0.29	79	0.771	0.03	− 0.19	0.26
	75%	2.74	79	0.023*	0.31	0.09	0.56
	62.5%	10.45	79	8.88*10^−16^***	1.17	0.88	1.53
	50%	12.49	79	1.93*10^−19^***	1.40	1.14	1.74
87.5%	75%	3.09	79	0.011*	0.35	0.12	0.59
	62.5%	11.56	79	9.12*10^−18^***	1.29	1.07	1.61
	50%	12.57	79	1.52*10^–19^***	1.41	1.15	1.73
75%	62.5%	10.30	79	1.48*10^−15^***	1.15	0.9	1.48
	50%	10.80	79	2.25*10^−16^***	1.21	0.95	1.56
62.5%	50%	2.38	79	0.040*	0.27	0.05	0.48

Paired sample t tests were performed to compare accuracies between completeness levels (t = test statistic, *df* degrees of freedom, *p* (corr.) = Holm-Bonferroni-corrected *p*-value, *d* = Cohen’s *d* and CI confidence interval).

Significance is indicated by *, ** or ***.

To test whether or not there is an equal weighting between the recognition of known versus new tactile patterns or whether there is a pattern completion or pattern separation bias for touch, we adopted the analysis of Vieweg et al.^22^ and calculated a bias score by subtracting the accuracy in the new conditions from the accuracy in the learned conditions for each completeness level separately (the score reveals a bias towards pattern completion for values > 0 and or a bias towards pattern separation for values < 0, Vieweg et al.^22^). Comparing the bias score to zero within each group reveals a significant pattern completion bias for both younger adults (M = 5.00, SD = 22.75, t(99) = 2.20, *p* = 0.0303, Cohen’s *d* = 0.22) and older adults (M = 11.75 SD = 28.48, t(99) = 4.13, *p* = 1.54*10^−4^, Cohen’s *d* = 0.41, see Fig. 3C), which presents with weak effect sizes. Forest plots uncover that in both younger and older adults, higher effect sizes for the pattern completion bias are obtained with higher stimulus completeness (see Fig. 3D).

We then tested whether or not the pattern completion bias for touch is more pronounced in older compared to younger adults, and whether this is dependent on the completeness level. A two-way ANOVA with the between-factor factors age (younger, older) and the within factors stimulus completeness (100%, 87.5%, 75%, 62.5%, 50%) on bias scores reveals low effect sizes for the main effect of age (η^2^ = 0.018) and for the interaction between age and stimulus completeness (η^2^ = 0.009, see Table 3 for full ANOVA results). Effect sizes calculated per completeness level reveal generally low effect sizes, but the highest value for the full completeness condition (100%: Cohen’s *d* = 0.39, 87.5%: Cohen’s *d* = 0.19, 75%: Cohen’s *d* = 0.36, 62.5%: Cohen’s *d* = − 0.03, 50%: Cohen’s *d* = 0.15). Also additional Bayesian analyses confirm that the likelihood for an age difference in pattern completion is generally low, but highest in the 100% completeness conditions (BF = 2.17, see Supplemental Material Table 3). This shows that both younger and older adults present with a pattern completion bias for touch, and that the extent to which this tactile pattern completion bias is higher in older compared to younger adults is low.

**Table 3:** Results of two-way ANOVA on bias with the factors age and completeness (experiment 2).

Effect	F	*DFn*	*DFd*	*p*	ŋ^2^
Age	1.21	*1*	*38*	0.279	0.018
Completeness	3.19	*4*	*152*	0.015*	0.035
Age * Completeness	0.80	*4*	*152*	0.526	0.009

Shown are the main effects of age and completeness, and the interaction effect between age and completeness.

F = test statistic, *DF* degree of freedom.

*p* = significance *p*-value, ŋ^2^ = effect size.

Significance is indicated by *.

### Older adults are specifically impaired in reaching peak performance in tactile memory tasks (experiments 1 and 2)

Whereas experiment 1 shows that older adults have only marginally lower precision in tactile memory performance compared to younger adults, experiment 2 reveals that older adults show only a marginally higher tactile memory bias than younger adults, even though both younger and older adults show a pronounced pattern completion bias for touch. The results of both experiments therefore uncover that with respect to precision and bias, tactile memories are surprisingly preserved in older age. Nevertheless, impairments in tactile memory performance are detected in older adults in both experiments. In experiment 1, older adults are particularly outperformed by younger adults in the easier experimental conditions (37.5%, 25%, and 12.5% similarity between ‘reference pattern’ and ‘new patterns’, where younger adults reach 95.3%, 88.0% and 98.7% correct performance, respectively, whereas older adults only reach 72.7%, 76.0% and 84.7%). One could therefore speculate that older adults are specifically impaired in reaching peak performance in tactile memory tasks. To test whether this is also true for experiment 2, we conducted a post-hoc analysis and calculated a three-way ANOVA with the between-factor age (younger, older) and the within-factors condition (learned, new) and stimulus completeness (100%, 87.5%, 75%, 62.5%, 50%) on accuracies. Results reveal an overall worse performance of older adults compared to younger adults (younger adults: M = 70.50, SD = 24.23, older adults: M = 60.13, SD = 23.14, F(1, 38) = 10.042, *p* = 0.003, η^2^ = 0.076, see Fig. 3A), and an interaction between age and stimulus completeness (F(4, 152) = 10.271, *p* = 2.18*10^−7^, η^2^ = 0.052). Both effects present with low effect sizes, but the inspection of effect sizes per completeness level reveals that the age difference is driven by complete or nearly complete stimuli (see Fig. 3B), where younger adults in particular outperform older adults (87.5% and 100% completeness levels, where younger adults reach 85.4% and 90.8%, whereas older adults only reach 70.4% and 66.0%). Taken together, in both experiments, older adults have specific deficits in reaching peak performance (i.e., accuracies > 85%).

## Discussion

We here introduce two new paradigms that were designed to better understand the cognitive mechanisms that underlie tactile memory retrieval, and their age-related changes. Our data reveals that older adults show only marginal impairments in tactile memory precision, tested via the ability to differentiate a learned tactile pattern that is passively presented to the fingertip from new tactile patterns with varying similarity (experiment 1). We further show that both younger and older adults present with a pattern completion bias for touch, evidenced by higher accuracies for detecting known compared to recognizing new tactile patterns (experiment 2). The pattern completion bias for touch was only marginally modulated by age. Instead, both experiments reveal that older adults have specific impairments in reaching peak performance > 85% in tactile memory experiments.

Memory performance is characterized by its precision and its bias^14^. Our first motivation was to test to what extent tactile memories are less precise in older compared to younger adults. To this end, in experiment 1, younger and older adults learned to recognize one tactile pattern (‘reference pattern’) with high degrees of accuracy. Subsequently, different tactile patterns were shown with varying levels of similarity to the ‘reference pattern’. If older adults had significantly less precise tactile memory representations compared to younger adults, one would expect that the 50% threshold at which new tactile patterns are correctly assigned as ‘new’ is significantly higher in older compared to younger adults. This hypothesis was not confirmed. The finding that aging decreases the precision of visual memories^15^ is not confirmed for the precision of tactile memories as additional Bayesian analyses also revealed that there is only anecdotal evidence for an age difference in precision. Research has shown that tactile representations in SI present with larger tuning widths in older compared to younger adults^19^. However, given the distance between the individual pins that formed one pattern (2.5 mm) was larger than the average 2-point discrimination threshold of older adults (1.3 mm^28^), subtle differences in the precision of sensory representations in SI did likely not influence our results, but may influence memory performance when tactile patterns are presented with higher spatial sampling density. At the level of spatial precision used here (i.e., 2.5 mm sampling density), the precision and “uniqueness” of tactile memory representations is only marginally reduced in older adults, and they are capable of memorizing subtle differences in tactile shape and form.

Our second motivation was to test if memory biases exist in touch, and to what extent they are more pronounced in older adults compared to younger adults. To this end, in experiment 2, a new group of younger and older adults first learned to discriminate three tactile patterns at the fingertip. In the actual experiment, they were presented either with one of the three learned patterns or with one of three new patterns, either shown complete or at a reduced completeness level (i.e., with fewer pins). We observed a bias in both younger and older adults to falsely assign new tactile patterns the status of known tactile patterns, whereas the reverse confusion was systematically smaller. This indicates a stronger contribution of pattern completion compared to pattern separation processes for tactile memory. During pattern completion, previously learned information is retrieved based on partial cues of this information^24,25^ and therefore subserves generalization and pattern recognition. A stored memory trace is reactivated from partial input to reproduce a similar representation compared to when the input information would be fully present^29^. The higher weightening of pattern completion over pattern separation indicates a bias towards favoring the perception of known over novel experiences, i.e., a higher weighting of the past compared to the present. This shift may allow an enhanced matching process between tactile perception and stored memory traces, which may allow recognizing a tactile object even when only partial tactile information is provided, for example when the hand is cold, the object is wet, or the texture is slightly different.

Because pattern completion and pattern separation are two cognitive processes that are assumed to compete with each other, the higher weighting of pattern completion indicates that the cognitive processes that subserve pattern separation may be less represented during tactile memory performance. The dentate gyrus, which is part of the hippocampal formation, has been linked to pattern separation^20,25,30,31^, with its projections to the hippocampal subfield CA3 allowing the storage of separated representations^23,32,33^. With the output of CA3 neurons being projected to CA1, a comparison between the perceptual input and its divergence from the retrieved information takes place. These representations can be later recalled even when partial input from the dentate gyrus is provided due to the projections from the entorhinal cortex to CA3. Impaired functioning of the dentate gyrus has been related to pattern completion biases^20^. One potential neuronal mechanism that could subserve a pattern completion bias for touch is hence the downregulation of the dentate gyrus and/or its connectivity patterns during tactile retrieval.

Usually, pattern completion subserves the ability to complete degraded (rather than complete) versions of sensory inputs. However, in experiment 2, the pattern completion bias was stronger for more complete patterns. There are three reasons why, for the sense of touch, a pattern completion bias may be stronger for inputs with more sensory information. First, the sense of touch is less precisely encoded compared to the sense of vision, and visual input usually overrides haptic input when both are conflicting^34^. A complete or nearly complete tactile pattern may therefore match the level of remembered precision of a visual image that is presented with noise or missing input. This hypothesis is supported by our observation in the piloting phase of the experiment that, other than in vision, participants have difficulties memorizing more than three tactile patterns at once, which is why we reduced the to-be-learned patterns to three in the final experiment. Second, whereas a large portion of the visual field is stimulated in visual experiments, here, we applied touch to a very small part of the body (the fingertip). Activating only a small portion of the receptor surface could trigger pattern completion processes similar to observing an image at a reduced field-of-view, or touching only parts of an object. Third, it has been suggested that pattern completion mechanisms of almost complete sensory information may rely on non-hippocampal sources^35^ and may be mediated by different mechanisms as the ones outlined above. Parietoinsular pathways that lead to the posteroventral insula have been associated to the long-lasting representations of tactual experiences^36^, and primary sensory cortices contribute to vivid offline representations of passive touch^37^. These mechanisms may follow different recruitment patterns than for the sense of vision.

The pattern completion bias was numerically more pronounced in older compared to younger adults, but this effect presented with a weak effect size. A numerically larger pattern completion bias in older adults was expected, because the connectivity between the dentate gyrus and CA3 is usually reduced in increasing age^38^. Aging rodents as well as older adults often show a stronger dominance of prior memories compared to new memories due to the reinforcement of the auto-associative network of CA3^22,39^. This process may increase the weighting towards pattern completion in older adults, leading to reduced performance of older adults to correctly recognize the complete or nearly complete new stimuli. In the present study, we did not assess functional connectivity patterns within the hippocampal formation, or other measures of functional or structural degeneration. It therefore remains open if preserved functioning of the outlined network is responsible for the weak effect size of the observed alterations. Future studies combining this paradigm with MRI and fMRI measurements may clarify this aspect.

One can only speculate about the neuronal mechanisms that may underlie this behavioral effect. With respect to alternative mechanisms that may explain the results, tactile mental imagery has been suggested to be either visually-driven or tactile-driven, with earlier studies proposing that tactile sensations might be transformed to either verbal or visual format when stored^40–42^, and visual frames of reference influence tactile shape recognition^43^. In the present experiment, the tactile patterns were associated with the names ‘pattern1’, ‘pattern2’, and ‘pattern3’. These verbal associations do not offer explicit links to shapes or visual images, but strategies relying on mental imagery of verbal or visual form may have been employed during this task. Other evidence suggests that mental imagery of tactile sensations elicits activation in SI, which suggests tactile-driven mental imagery^44^, and a role of SI in tactile working memory^45,46,^ as well as the storage and retrieval of sensory memories^47^ has been proposed. Finally, a critical role of the association cortex for shape recognition and sensory memory has been evidenced^48^.

We conducted post-hoc analyses to understand age-related impairments in tactile memory in greater detail. Those reveal that older adults specifically present with a lower peak performance in both experiments. More precisely, in relatively easy experimental conditions, where new tactile patterns of low similarity to the learned one have to be detected (experiment 1), or complete versions of learned and new tactile patterns have to be distinguished (experiment 2), older adults have difficulties to reach peak performance > 85%, whereas such performance levels are easily reached by younger adults. This cannot be due to differences in reaction times as participants were allowed to answer at their own pace. We also controlled for individual differences in sensory perception thresholds by adjusting stimulation strength to the individual threshold. Rather, these results point towards age-related impairments in recruiting memory networks over an extended time period that allow steady peak performance, including the effective interplay between the hippocampus, the dentate gyrus, SI and association cortex, and other potentially involved networks, for example networks mediating attention^49^. However, because we did not conduct MRI measurements, it remains open which brain networks underlie the observed effects, and the potential role of neurodegeneration needs to be determined.

With respect to further study limitations, it is worth noting that in our design, encoding and retrieval could not fully be differentiated. During pattern completion, the stored memory trace is activated even if only partial information is provided. Therefore, the process of encoding new information overlaps with the retrieval of stored information^50^, which limits the insights that can be derived from purely behavioral designs.

Taken together, we here introduce two novel paradigms to study fine-grained memory of tactile patterns presented to the fingertip. Our results indicate that both younger and older adults show a higher weighting of past compared to present tactile experiences. This shift may allow an enhanced matching process between tactile perception and stored memory traces, which may allow recognizing a tactile object even when only partial tactile information is provided, for example when the hand is cold or the object is wet. Age-related differences in the precision of tactile memories were marginal, but older adults show specific difficulties in reaching peak performance, which points towards age-related impairments in recruiting memory networks over an extended time period that allow steady peak performance.

## Methods

### Participants

We recruited younger adults between 18 and 30 years, and older adults above 65 years of age. This is the same age range chosen by previous studies on tactile and cognitive aging^50–55^. In experiment 1, n = 43 right-handed participants took part, of whom n = 15 younger adults (26.33 ± 2.35, 8 female) and n = 15 older adults (68.93 ± 6.42, 7 female) successfully completed the training. In experiment 2, a new group of n = 52 right-handed participants took part, of whom n = 20 younger adults (25.3 ± 3.04 years, 10 female), and n = 20 older adults (71.65 ± 5.27 years, 10 female) successfully completed the training. The Montreal Cognitive Assessment (MoCA)^56^ was used as a screening tool to exclude the possibility of Mild Cognitive Impairment (MCI) that could influence memory function^57^. The cut-off score used here was 23/30 points. Although the cut-off score of 26 is often used, participants with the scores 23–25 were included because this ensures that cognitively normal individuals will not falsely be identified as having MCI^58^. Three older adults (n = 1 female) were excluded because they scored lower than 23 points in the MoCA. None of them suffered from any neurological or psychiatric illness, and none of them took medication that could influence the function of the central nervous system. They also did not experience injuries on their hands or fingers in the past. We did not invite professional sportsmen/-women and musicians, as they could show differential performance due to excessive hand usage and consequently injuries or permanent modifications of the glabrous skin as well as different cortical representations^59–61^. All participants provided written informed consent to take part in the experiment, after being informed that their personal data would remain private and protected. They had the right to end the testing if and whenever they wanted without any cost. The study received approval of the Ethics Committee of the Otto-von-Guericke University Magdeburg, Germany, and all experiments were performed in accordance with relevant guidelines and regulations. Participants were reimbursed for their participation with a monetary compensation of 7.50€/h.

### Tactile Patterns

For tactile stimulation, piezoelectric vibrotactile stimulation was used and applied to participants' right fingertips using the QuearoSys Piezostimulator (Piezostimulator, Quaerosys, St. Johann, Germany) controlled via a Thinkpad notebook and Matlab (R2015b) via the Psychtoolbox (last update: 07/02/2013, see Fig. 1A), the same as several previous studies^19,62^. Passive tactile stimulation was applied to the right index fingertip via a stimulation module that was fixated at the fingertip via a tape. The stimulation module consisted of a 4 × 4 pin matrix (i.e., n = 16 pins) with 2.5 mm spacing between each pin. Each pin of the matrix was controlled separately and is able to reach 4096 different heights between 0 mm and 1.5 mm with a timing accuracy of 0.5 ms.

For experiment 1, 10 tactile patterns were created with this matrix, where for each pattern, 8 pins were used. For experiment 2, 9 new tactile patterns were created with this matrix, where for each complete pattern, 8 pins were used. In order to present each tactile pattern to the fingertip, the respective pins vibrated at 10 Hz frequency using a sinusoidal amplitude and smoothed stimulus onsets and offsets. This frequency was chosen to optimize fine-grained tactile perception at the fingertip via SA1 fibers^63^. Each pattern was presented for 1000 ms. Although increased display times of tactile stimuli improve recognition performance ^50^, a stimulation time of 1000 ms was chosen because longer stimulation durations (e.g., 3000 ms) are usually chosen for studies on tactile mental imagery^64^, a process that we did not target, and a 1000 ms duration has been shown to lead to similar tactile threshold shifts in younger and older adults^65^. Note that the tactile patterns used in experiment 2 had a symmetrical shape but different orientations so that the similarity of the applied pattern was comparable across conditions.

### Tactile threshold task

Before both experiments started, the threshold to detect one pin on the skin surface was estimated using the same stimulator as introduced above. The detection threshold was then used to adapt the stimulation amplitude individually. For this task, one pin of the 4 × 4 array (in the middle of the array) was used, and presented for the duration of 1000 ms. On screen, the participant saw the numbers ‘1’ and ‘2’, and had to decide in which interval the stimulus was perceived (two-alternative forced-choice test)^66^. The stimulation occurred randomly either in the first or second interval. Responses were given at a self-determined pace. An adaptive staircase procedure (3-down, 1-up, where one step was programmed as 80 steps within the Piezo Stimulator module) was used to determine the individual detection threshold. If after a minimum of 40 trials the amplitude did not vary from the mean more than two times the step size, the stimulation ended automatically and the last amplitude was defined as the detection threshold. This value was then multiplied by 2 so that the learning experiment took place at the twofold detection threshold. This procedure took approximately 15 min. There was a break after the tactile detection task was completed and before the Experiment started.

### Tasks

Experiment 1 was completed in one session and lasted approximately 30 to 45 min. First, one tactile pattern was learned in the learning phase. This pattern is referred to as ‘Reference’ (see Fig. 1C). In the learning phase, each participant was presented with the Reference for 10 times in a row. Then, participants had to discriminate the Reference pattern from two new tactile patterns via a two-alternative forced-choice task (‘learned’ versus ‘new’). The two new tactile patterns had only 1 or 2 overlapping pins, respectively, with the reference pattern and were therefore clearly different. The learning phase was successfully completed when each tactile pattern was identified correctly for three times in a row. If this was not achieved, the participant was allowed to repeat learning phase 2 for two times. If the criterion was still not achieved, the participant was not allowed to proceed to the experimental phase. This was the case for n = 1 younger adult and n = 2 older adults, who did not fulfill this criterion and terminated the experiment after the learning phase.

In the experimental phase, 7 new tactile patterns were presented together with the Reference pattern in a randomized order. The similarity between the 7 new tactile patterns and the Reference pattern was systematically modulated: the new patterns had either 1, 2, 3, 4, 5, 6 or 7 overlapping pins with the reference pattern (corresponding to 12.5—87.5% similarity, see Fig. 1C). Each pattern was presented 10 times, resulting in 80 trials altogether. In the experimental phase, participants had to respond via a two-alternative forced-choice task (‘learned’ versus ‘new’). If participants completed the learning phase successfully, but failed to recognize the reference pattern in more than 60% of the cases in the experimental phase, they were allowed to undergo the learning phase a maximum of two additional times. This was the case for n = 4 younger adults and n = 6 older adults.

Experiment 2 lasted approximately 45 to 75 min. In this experiment, three tactile patterns needed to be learned in learning phase 1 (‘Learned’ tactile patterns, see Fig. 1). The three patterns were first presented to participants’ fingertips for 10 times each together with visual labels on screen (i.e., ‘pattern 1’, ‘pattern 2’, ‘pattern 3’, 2 s pause between stimulus presentations), and were then presented 10 times in random order together with the same visual labels. Then, the three Learned patterns were randomly presented without the visual labels, and participants had to identify each pattern via a three-alternative forced-choice task (possible answers: ‘pattern 1’, ‘pattern 2’, ‘pattern 3’) via left hand button presses. Participants received feedback after each trial allowing them to improve performance. Learning phase 1 automatically ended when each pattern was correctly identified 3 times in a row. If this was not achieved after each pattern was presented maximally 10 times, the participant was allowed to repeat learning phase 1 for three times. If the criterion was still not achieved, the participant was not allowed to proceed to learning phase 2. This was the case for n = 7 older adults, who did not fulfill this criterion and terminated the experiment after learning phase 1.

In learning phase 2, the three Learned tactile patterns again had to be identified (as in learning phase 1), but here, also three new tactile patterns were presented (‘New’ tactile patterns, see Fig. 1). All six tactile patterns were presented in random order, and with the same stimulation frequency and intertrial interval as before. Participants had to solve a four-alternative forced-choice task (possible answers: ‘pattern1’, ‘pattern2’, ‘pattern3’, ‘new’). Participants received feedback after each trial to allow them to improve in learning phase 2. Learning phase 2 automatically ended when each Learned pattern was correctly identified three times in a row. If this was not achieved after each pattern was presented maximally 10 times, the participant was allowed to repeat learning phase 2 for three times. If the criterion was still not achieved, the participant was not allowed to proceed to the experimental phase. This was the case for n = 5 older adults, who did not fulfill this criterion and terminated the experiment after learning phase 2.

After these two training phases, the actual experiment 2 started. The experimental phase was similar to learning phase 2, except that a different set of New tactile patterns was used, and that all patterns (learned and new) were presented at varying completeness levels: 100% (8/8 pins), 87.5% (7/8 pins), 75% (6/8 pins), 62.5% (5/8 pins), and 50% (4/8 pins) (see Fig. 1). Completeness levels were chosen in a way to ensure that every incomplete version of each pattern could only be completed to one other pattern (i.e., it was not possible to complete one incomplete pattern into more than one complete pattern). In addition, completeness levels were chosen in a way to ensure that within the Learned patterns and within the New patterns, as well as between categories, the grade of pin similarity would not be significantly different. Similarity was here defined as the mean number of different pins when comparing the patterns of each category with the patterns of the same/different category across all completeness levels. Each of these five completeness levels was presented four times, resulting in 20 trials per pattern, and a total of 120 experimental trials. As in learning phase 2, participants had to solve a four-alternative forced-choice task (possible answers: ‘pattern1’, ‘pattern2’, ‘pattern3’, ‘new’). To make sure that the name of the patterns did not affect performance, the associations between patterns and names were changed in half of the participants of each age group. Participants were provided with the respective completeness level of each trial on screen via a bar. This allowed them to estimate how many pins would need to be mentally ‘completed’. After the response, participants rated their confidence about their decision on a scale from 1 (‘not at all confident’) to 4 (‘very confident’). No feedback was provided. After 60 trials, there was a one minute break to prevent fatigue effects. The duration of the experimental phase was approximately 20 min.

## Analyses

RStudio 2022.07.0 and the R packages ‘rstatix’, and ‘tidyverse’ were used for statistical analyses and R package ‘ggpubr’ was used for data visualization. Data were tested for the assumption of normal distribution with the Shapiro Wilk’s test of normality (*p* > 0.05) and the assumption of homoscedasticity with the Levene’s test of homogeneity of variance (*p* > 0.05). If homogeneity of variance was not given according to Levene’s test of homogeneity of variance, the Welch’s t-test was used. When the majority of conditions were normally distributed, but single conditions were not normally distributed, t-tests were used because simulation studies showed that t-tests are relatively robust against minor violations of the normal distribution assumption (e.g.^67^).

### Variables

Both in experiment 1 and in experiment 2, we calculated the accuracy to estimate the relative proportion of the number of times the participant correctly recognized the shown tactile pattern out of the total number of presentations of the corresponding tactile pattern in percentage. The accuracy was calculated for each participant and each tactile pattern separately and served as a measure of participants' performance in the respective task.

In experiment 2, we additionally calculated a previously published bias score to estimate pattern completion and pattern separation biases in younger and older adults^22^. For this purpose, we subtracted the accuracy for new patterns from the accuracy of learned patterns for all completeness levels separately^22^. According to Vieweg et al.^22^, positive values in this bias score indicate a bias towards pattern completion, whereas negative values indicate a bias towards pattern separation.

### Statistical analyses experiment 1

To estimate if participants could successfully solve the task, two-sided one-sample t-tests were computed to compare the accuracy of younger and older adults to chance level (i.e., to 50%). T-tests were Holm-Bonferroni corrected at a significance threshold of *p* < 0.05. To compare the accuracy of younger and older adults for each tactile pattern, unpaired two-sided Welch’s t-tests were performed and Holm-Bonferroni corrected (*p* < 0.05). In addition, Bayesian independent samples t-test with the between-subjects factor age was performed to investigate the learning effect of age groups. Bayes factor favoring the alternative hypothesis was calculated using 50% thresholds of the accuracy and the R package ‘BayesFactor’. Bayes factor is a method of hypothesis testing in which the probability for one model under the condition of an alternative model given the observed data is computed. To be precise, the Bayes factor is calculated by the ratio of the density under the distribution of the null model to the density under the distribution of the alternative model. Hence, prior assumptions about the distribution are crucial: The R package ‘BayesFactor’ relies on the Cauchy distribution that is similar to a normal distribution^68^. The resulting value indicates how much more likely the alternative hypothesis is compared to the null hypothesis. Thus, the Bayes factor quantifies the degree of evidence for one hypothesis relative to another given the observed data^69^. A Bayes factor above 1/3 is considered as anecdotal evidence, above 3 as some evidence and above 10 as strong evidence^70^.

50% thresholds were calculated using binary logistic regression as stated in Kuehn et al.^71^. Accuracies were plotted against all new tactile patterns (87.5%, 70%, 62.5%, 50%, 37.5%, 25%, and 12.5%) for each individual and were fitted a sigmoid curve using the Statistics and Machine Learning toolbox in Matlab (MATLAB_R2021a). In order to find out if the logistic regression model fits the data without further predictors (null model), deviance of the fitted model was compared with the deviance of the null model and p-values were calculated with likelihood ratio test. A significance level of 20% was determined as a cut-off criterion, which entails that goodness-of-fit for the logistic regression model is adequate and the null-model can be rejected when p-value is lower than 0.20. In previous work on the tactile system, a significance level of 15% was used with this function^71^. A more lenient threshold was used here given the curve fitting was performed on memorized tactile items rather than on actually perceived tactile items, which includes higher levels of noise for each condition. This was found for N = 17 subjects (N_young_ = 9). 50% thresholds were extracted from the logistic regression models of the individuals with p-value lower 0.20 and used as dependent variables for Bayesian t-test.

The mean %responses ‘new’ were plotted using the spline function from the Matlab Curve Fitting Toolbox, and the 50% thresholds were calculated based on each individual participant and the mean group responses. Note that the new tactile pattern with a similarity of 87.5% to the reference pattern was assigned to the x-value 2, the new tactile pattern with a similarity of 70% to the x-value 3 and so on. Tactile detection thresholds were compared using the Welch’s test because homoscedasticity was not given.

### Statistical Analyses experiment 2

To estimate if participants could successfully solve the task, two-sided one-sample t-tests were computed to compare mean accuracy scores of all completeness levels to chance level (i.e. to 25%). T-tests were Holm-Bonferroni corrected at a significance threshold of *p* < 0.05 for younger and older adults separately. To compare bias scores against zero, two two-tailed one-sample t-tests were computed with an Holm-Bonferroni adjusted significance threshold of *p* < 0.05. After examining the assumptions (see above), a three-way mixed ANOVA with the between factor age and the two within factors condition and stimulus completeness was calculated. Dependent Welch’s t-tests were used as post-hoc tests for the main effects of the within factors, and independent Welch’s t-tests for the main effect of the between factors. Pairwise Welch’s t-tests were conducted for interaction effects. All multiple comparisons were Holm-Bonferroni corrected (*p* < 0.05). Cohens’ *d* was used to estimate the effect sizes. Tactile detection thresholds were compared using independent two-tailed t-tests and Cohen’s *d* as effect size.

### Supplementary Information

Supplementary Tables.

## Data Availability

Data will be made available upon formal request to Lilith-Sophie Lange.

## References

[1] Klatzky RL, Lederman SJ, Metzger VA (1985). Identifying objects by touch: An ‘expert system’. Percept. Psychophys..

[2] Gentsch A, Kuehn E (2022). Clinical manifestations of body memories: The impact of past bodily experiences on mental health. Brain Sci..

[3] Kuehn E (2018). Embodiment in the aging mind. Neurosci. Biobehav. Rev..

[4] Boyle E, Aparicio AM, Kaye J, Acker M (1975). Auditory and visual memory losses in aging populations. J. Am. Geriatr. Soc..

[5] Merhav M, Wolbers T (2019). Aging and spatial cues influence the updating of navigational memories. Sci. Rep..

[6] Blachstein H, Vakil E (2016). Verbal learning across the lifespan: An analysis of the components of the learning curve. Neuropsychol. Dev. Cogn. B Aging Neuropsychol. Cogn..

[7] Ballesteros S, Reales JM (2004). Intact haptic priming in normal aging and Alzheimer’s disease: Evidence for dissociable memory systems. Neuropsychologia.

[8] Ferreira CD (2021). Long-term memory of haptic and visual information in older adults. Neuropsychol. Dev. Cogn. B Aging Neuropsychol. Cogn..

[9] Larsson M, Bäckman L (1998). Modality memory across the adult life span: Evidence for selective age-related olfactory deficits. Exp. Aging Res..

[10] Riege WH, Metter EJ, Williams MV (1980). Age and hemispheric asymmetry in nonverbal tactual memory. Neuropsychologia.

[11] Riege WH, Inman V (1981). Age differences in nonverbal memory tasks. J. Gerontol..

[12] Lazarus JA, Haynes JM (1997). Isometric pinch force control and learning in older adults. Exp. Aging Res..

[13] Dunn W (2015). Measuring change in somatosensation across the lifespan. Am. J. Occup. Ther..

[14] Ekstrom AD, Yonelinas AP (2020). Precision, binding, and the hippocampus: Precisely what are we talking about?. Neuropsychologia.

[15] Esfahan SM, Nili MHHK, Hatami J, Sanayei M, Rezayat E (2023). Aging decreases the precision of visual working memory. Neuropsychol. Dev. Cogn. B Aging Neuropsychol. Cogn..

[16] Bennett CR, Loomis JM, Klatzky RL, Giudice NA (2017). Spatial updating of multiple targets: Comparison of younger and older adults. Mem. Cognit..

[17] Pertzov Y, Heider M, Liang Y, Husain M (2015). Effects of healthy ageing on precision and binding of object location in visual short term memory. Psychol. Aging.

[18] Koen JD (2022). Age-related neural dedifferentiation for individual stimuli: An across-participant pattern similarity analysis. Neuropsychol. Dev. Cogn. B. Aging Neuropsychol. Cogn..

[19] Liu P (2021). The organizational principles of de-differentiated topographic maps in somatosensory cortex. Elife.

[20] Baker S (2016). The human dentate gyrus plays a necessary role in discriminating new memories. Curr. Biol..

[21] Grande X (2019). Holistic recollection via pattern completion involves hippocampal subfield CA3. J. Neurosci..

[22] Vieweg P, Stangl M, Howard LR, Wolbers T (2015). Changes in pattern completion—A key mechanism to explain age-related recognition memory deficits?. Cortex.

[23] Yassa MA (2011). Pattern separation deficits associated with increased hippocampal CA3 and dentate gyrus activity in nondemented older adults. Hippocampus.

[24] Marr D (1971). Simple memory: a theory for archicortex. Phil. Trans R. Soc. Lond B Biol. Sci..

[25] Paleja M, Spaniol J (2013). Spatial pattern completion deficits in older adults. Front. Aging Neurosci..

[26] Thornbury JM, Mistretta CM (1981). Tactile sensitivity as a function of Age. J. Gerontol..

[27] Liu, P. *et al.* A layer-specific model of cortical sensory aging. 12.01.567841 Preprint at 10.1101/2023.12.01.567841 (2023).

[28] Rolls ET (2016). Pattern separation, completion, and categorisation in the hippocampus and neocortex. Neurobiol. Learn. Mem..

[29] Berron D (2016). Strong evidence for pattern separation in human dentate gyrus. J. Neurosci..

[30] Kirwan CB (2012). Pattern separation deficits following damage to the hippocampus. Neuropsychologia.

[31] Bakker A, Kirwan CB, Miller M, Stark CEL (2008). Pattern separation in the human hippocampal CA3 and dentate gyrus. Science.

[32] Neunuebel JP, Knierim JJ (2014). CA3 retrieves coherent representations from degraded input: direct evidence for CA3 pattern completion and dentate gyrus pattern separation. Neuron.

[33] Myers CE, Scharfman HE (2011). Pattern separation in the dentate gyrus: a role for the CA3 backprojection. Hippocampus.

[34] Ernst MO, Banks MS (2002). Humans integrate visual and haptic information in a statistically optimal fashion. Nature.

[35] Theves S, Grande X, Düzel E, Döller C (2022). Handbook of the human memory.

[36] Bonda E, Petrides M, Evans A (1996). Neural systems for tactual memories. J. Neurophysiol..

[37] Kuehn E, Haggard P, Villringer A, Pleger B, Sereno MI (2018). Visually-driven maps in area 3b. J. Neurosci..

[38] Holden HM, Gilbert PE (2012). Less efficient pattern separation may contribute to age-related spatial memory deficits. Front. Aging Neurosci..

[39] Wilson IA, Gallagher M, Eichenbaum H, Tanila H (2006). Neurocognitive aging: Prior memories hinder new hippocampal encoding. Trends Neurosci..

[40] Gallace A (2013). Multisensory imagery.

[41] Mahrer P, Miles C (2002). Recognition memory for tactile sequences. Memory.

[42] Picard D, Monnier C (2009). Short-term memory for spatial configurations in the tactile modality: A comparison with vision. Memory.

[43] Heller MA (1989). Tactile memory in sighted and blind observers: the influence of orientation and rate of presentation. Perception.

[44] Schmidt TT, Blankenburg F (2019). The somatotopy of mental tactile imagery. Front. Hum. Neurosci..

[45] Harris JA, Harris IM, Diamond ME (2001). The topography of tactile working memory. J. Neurosci..

[46] Harris JA, Miniussi C, Harris IM, Diamond ME (2002). Transient storage of a tactile memory trace in primary somatosensory cortex. J. Neurosci..

[47] Harris JA, Petersen RS, Diamond ME (2001). The cortical distribution of sensory memories. Neuron.

[48] Dahl MJ (2019). Rostral locus coeruleus integrity is associated with better memory performance in older adults. Nat. Hum. Behav..

[49] Craig JC (1983). Some factors affecting tactile pattern recognition. Int. J. Neurosci..

[50] Reuter E-M, Voelcker-Rehage C, Vieluf S, Godde B (2014). Effects of age and expertise on tactile learning in humans. Eur. J. Neurosci..

[51] Dinse HR (2005). Improving human haptic performance in normal and impaired human populations through unattended activation-based learning. ACM Trans. Appl. Percep..

[52] Stevens JC, Alvarez-Reeves M, Dipietro L, Mack GW, Green BG (2003). Decline of tactile acuity in aging: a study of body site, blood flow, and lifetime habits of smoking and physical activity. Somatosens. Mot. Res..

[53] Coolin A, Bernstein DM, Thornton AE, Thornton WL (2014). Age differences in hindsight bias: The role of episodic memory and inhibition. Exp. Aging Res..

[54] Murphy NA, Isaacowitz DM (2008). Preferences for emotional information in older and younger adults: A meta-analysis of memory and attention tasks. Psychol. Aging.

[55] Dinse HR (2006). Tactile coactivation resets age-related decline of human tactile discrimination. Ann. Neurol..

[56] Nasreddine ZS (2005). The Montreal Cognitive Assessment, MoCA: A brief screening tool for mild cognitive impairment. J. Am. Geriatr. Soc..

[57] Petersen RC (2018). Practice guideline update summary: Mild cognitive impairment: Report of the guideline development, dissemination, and implementation subcommittee of the american academy of neurology. Neurology.

[58] Luis CA, Keegan AP, Mullan M (2009). Cross validation of the montreal cognitive assessment in community dwelling older adults residing in the southeastern US. Int. J. Geriatr. Psychiatry.

[59] Elbert T, Pantev C, Wienbruch C, Rockstroh B, Taub E (1995). Increased cortical representation of the fingers of the left hand in string players. Science.

[60] Ragert P, Schmidt A, Altenmüller E, Dinse HR (2004). Superior tactile performance and learning in professional pianists: Evidence for meta-plasticity in musicians. Eur. J. Neurosci..

[61] Schwenkreis P (2007). Assessment of sensorimotor cortical representation asymmetries and motor skills in violin players. Eur. J. Neurosci..

[62] Doehler J (2023). The 3D Structural Architecture of the Human Hand Area Is Nontopographic. J. Neurosci..

[63] Kim SS, Sripati AP, Bensmaia SJ (2010). Predicting the timing of spikes evoked by tactile stimulation of the hand. J. Neurophysiol..

[64] Nierhaus T (2023). Content representation of tactile mental imagery in primary somatosensory cortex. eNuro.

[65] Frisina RD, Gescheider GA (1977). Comparison of child and adult vibrotactile thresholds as a function of frequency and duration. Percep. Psychophys..

[66] Gescheider GA, Beiles EJ, Checkosky CM, Bolanowski SJ, Verrillo RT (1994). The effects of aging on information-processing channels in the sense of touch: II. Temporal summation in the P channel. Somatosens Mot. Res..

[67] Poncet A, Courvoisier DS, Combescure C, Perneger TV (2016). Normality and sample size do not matter for the selection of an appropriate statistical test for two-group comparisons. Methodology.

[68] Morey, R. D. *et al.* Package ‘Bayes Factor’ 0.9.12–4.6. https://cran.r-project.org/web/packages/BayesFactor/BayesFactor.pdf, (2023).

[69] Schmalz X, Biurrun Manresa J, Zhang L (2023). What is a Bayes factor?. Psychol. Methods.

[70] Dienes Z (2014). Using Bayes to get the most out of non-significant results. Front. Psychol..

[71] Kuehn E, Doehler J, Pleger B (2017). The influence of vision on tactile Hebbian learning. Sci. Rep..

