# Supplementary material for: Tactile memory impairments in younger and older adults
Source: Sci Rep. 2024 May 23;14:11766. doi: 10.1038/s41598-024-62683-y (PMC11116509; doi:10.1038/s41598-024-62683-y)
Supplement: Supplementary file 1 — Supplementary Tables. [file 41598_2024_62683_MOESM1_ESM.pdf]

**Supplemental Material**

# Tactile Memory Impairments in Younger and Older Adults

Lilith-Sophie Lange[1], Anastasia Chrysidou[1], Peng Liu[2,3], * Esther Kuehn[1,2,3]

[1] Institute for Cognitive Neurology and Dementia Research (IKND), Medical Faculty, Otto-von-Guericke University Magdeburg, Leipziger Straße 44, 39120 Magdeburg, Germany

[2] Hertie Institute for Clinical Brain Research, Otfried-Müller Straße 27, 72076 Tübingen, Germany

[3] German Center for Neurodegenerative Diseases (DZNE), Otfried-Müller Straße 23, 72076 Tübingen, Germany

**Corresponding Author**

Esther Kühn (Prof. Dr.), Esther.Kuehn@uni-tuebingen.de

Hertie Institute for Clinical Brain Research
Eberhard Karls University Tübingen
Otfried-Müller-Straße 27
72076 Tübingen
Germany

| Experiment 1 | | | | | | | | | |
|---|---|---|---|---|---|---|---|---|---|
| | Tactile pattern | Accuracy | | | | | | *CI* | |
| Age | (% similarity) | Mean | SD | t | *df* | *p* (corr.) | *d* | *Lower* | *Upper* |
| Younger | Reference | 76.67 | 12.34 | 8.37 | 14 | $4.860*10^{-6}$*** | 2.16 | *1.74* | *3.53* |
| | 87.5% | 35.33 | 32.70 | -1.73 | 14 | 0.312 | -0.45 | *-1.41* | *0.09* |
| | 70% | 47.33 | 34.32 | -0.30 | 14 | 0.768 | -0.08 | *-0.71* | *0.44* |
| | 62.5% | 62.00 | 34.06 | 1.36 | 14 | 0.388 | 0.35 | *-0.15* | *1.25* |
| | 50% | 70.67 | 28.90 | 2.76 | 14 | 0.060 | 0.72 | *0.16* | *2.26* |
| | 37.5% | 95.33 | 9.16 | 19.18 | 14 | $1.330*10^{-10}$*** | 4.95 | *3.19* | *19.11* |
| | 25% | 88.00 | 19.35 | 7.61 | 14 | $1.225*10^{-5}$*** | 1.96 | *1.14* | *6.45* |
| | 12.5% | 98.67 | 3.52 | 53.57 | 14 | $1.072*10^{-16}$*** | 13.83 | *9.56* | *19.11* |
| Older | Reference | 81.33 | 14.08 | 8.62 | 14 | $4.536*10^{-6}$*** | 2.23 | *1.63* | *3.76* |
| | 87.5% | 33.33 | 18.77 | -3.43 | 14 | $8.400*10^{-3}$** | -0.89 | *-1.68* | *-0.41* |
| | 70% | 31.33 | 25.03 | -2.88 | 14 | 0.0119* | -0.75 | *-1.79* | *-0.25* |
| | 62.5% | 72.00 | 21.78 | 3.91 | 14 | $6.240*10^{-3}$** | 1.01 | *0.47* | *2.41* |
| | 50% | 73.33 | 24.98 | 3.62 | 14 | $8.400*10^{-3}$** | 0.93 | *0.45* | *2.11* |
| | 37.5% | 72.67 | 21.20 | 4.14 | 14 | $5.676*10^{-3}$** | 1.07 | *0.63* | *1.89* |
| | 25% | 76.00 | 24.44 | 4.12 | 14 | $9.700*10^{-3}$** | 1.07 | *0.44* | *3.65* |
| | 12.5% | 84.67 | 23.26 | 5.77 | 14 | $3.563*10^{-4}$*** | 1.49 | *0.71* | *3.97* |

| Experiment 2 | | | | | | | | | | |
|---|---|---|---|---|---|---|---|---|---|---|
| | | Tactile pattern | Accuracy | | | | | | *CI* | |
| | Age | (% similarity) | Mean | SD | t | *df* | *p* (corr.) | *d* | *Lower* | *Upper* |
| Learned | Younger | 100% | 94.17 | 5.48 | 56.50 | *19* | $6.200*10^{-22}$*** | *12.63* | *10.28* | *17.64* |
| | | 87.5% | 90.83 | 10.78 | 27.31 | *19* | $4.160*10^{-16}$*** | *6.11* | *4.23* | *11.78* |
| | | 75% | 77.08 | 17.91 | 13.01 | *19* | $1.971*10^{-10}$*** | *2.91* | *2.02* | *4.67* |
| | | 62.5% | 55.42 | 16.06 | 8.47 | *19* | $1.418*10^{-7}$*** | *1.89* | *1.27* | *3.18* |
| | | 50% | 47.50 | 17.96 | 5.60 | *19* | $2.100*10^{-5}$*** | *1.25* | *0.84* | *2.14* |

| | | | | | | | | | | |
|---|---|---|---|---|---|---|---|---|---|---|
| | Older | 100% | 75.42 | 16.10 | 14.00 | *19* | $9.200*10^{-11}$*** | *3.13* | *2.36* | *4.78* |
| | | 87.5% | 79.58 | 19.02 | 12.83 | *19* | $3.296*10^{-10}$*** | *2.87* | *2.14* | *4.77* |
| | | 75% | 76.25 | 18.98 | 12.07 | *19* | $6.990*10^{-10}$*** | *2.70* | *2.13* | *3.81* |
| | | 62.5% | 51.25 | 14.63 | 8.02 | *19* | $3.200*10^{-7}$*** | *1.79* | *1.39* | *2.63* |
| | | 50% | 47.50 | 13.00 | 7.74 | *19* | $3.200*10^{-7}$*** | *1.73* | *1.24* | *2.68* |
| New | Younger | 100% | 87.5 | 13.92 | 20.08 | *19* | $1.480*10^{-13}$*** | *4.49* | *3.06* | *9.06* |
| | | 87.5% | 80.00 | 18.22 | 13.50 | *19* | $1.380*10^{-10}$*** | *3.02* | *2.14* | *5.14* |
| | | 75% | 74.17 | 16.42 | 13.39 | *19* | $1.380*10^{-10}$*** | *2.99* | *2.51* | *4.07* |
| | | 62.5% | 50.83 | 22.28 | 5.19 | *19* | $1.052*10^{-4}$*** | *1.16* | *0.75* | *1.79* |
| | | 50% | 47.50 | 23.74 | 4.24 | *19* | $4.440*10^{-4}$*** | *0.95* | *0.55* | *1.61* |
| | Older | 100% | 56.67 | 24.87 | 5.69 | *19* | $5.190*10^{-5}$*** | *1.27* | *0.98* | *1.78* |
| | | 87.5% | 61.25 | 26.80 | 6.05 | *19* | $3.240*10^{-5}$*** | *1.35* | *0.89* | *2.29* |
| | | 75% | 62.50 | 21.88 | 7.66 | *19* | $1.575*10^{-6}$*** | *1.71* | *1.32* | *2.43* |
| | | 62.5% | 47.92 | 21.10 | 4.86 | *19* | $2.180*10^{-4}$*** | *1.09* | *0.73* | *1.71* |
| | | 50% | 42.92 | 17.99 | 4.45 | *19* | $2.720*10^{-4}$*** | *1.00* | *0.60* | *1.62* |

**Supplemental Material - Table 1. Above-chance level performance of younger and older adults (experiment 1, experiment 2).** Shown are mean accuracies (mean) and standard deviations (SD) to correctly recognize the ‘reference pattern’ and ‘new patterns’ compared to chance level (50%) for younger and older adults separately (experiment 1); shown are also mean accuracies (mean) and standard deviations (SD) to correctly recognize the ‘learned patterns’ and the ‘new patterns’ compared to chance level (25%) for younger and older adults separately (experiment 2).One sample t tests were calculated to compare between the chance level and the mean accuracies (t = test statistic, *df* = degrees of freedom, *p* (corr.) = Holm-Bonferroni-corrected *p*-value, *d* = Cohen’s *d* and CI = confidence interval). Significance is indicated by *, ** or ***.

| Comparison between similarity levels | | t | *df* | *p* (corr.) | Cohen's *d* | *CI* | |
|---|---|---|---|---|---|---|---|
| | | | | | | *Lower* | *Upper* |
| 87.5% | 70% | 0.85 | 29 | 0.802 | 0.16 | *-0.21* | *0.54* |
| | 62.5% | 4.10 | 29 | 0..004** | 0.75 | *0.33* | *1.59* |
| | 50% | 5.05 | 29 | $3.28*10^{-4}$*** | 0.92 | *0.50* | *1.61* |
| | 37.5% | 9.21 | 29 | $8.24*10^{-9}$*** | 1.68 | *1.27* | *2.37* |
| | 25% | 6.36 | 29 | $9.65*10^{-6}$*** | 1.16 | *0.73* | *1.88* |
| | 12.5% | 9.26 | 29 | $7.79*10^{-9}$*** | 1.69 | *1.18* | *2.52* |
| 70% | 62.5% | 3.37 | 29 | 0.022* | 0.6 | *0.24* | *1.21* |
| | 50% | 4.02 | 29 | 0.004** | 0.73 | *0.30* | *1.37* |
| | 37.5% | 8.12 | 29 | $1.07*10^{-7}$*** | 1.48 | *1.07* | *2.06* |
| | 25% | 6.39 | 29 | $9.33*10^{-6}$*** | 1.16 | *0.75* | *1.80* |
| | 12.5% | 8.23 | 29 | $8.44*10^{-8}$*** | 1.50 | *1.07* | *2.36* |
| 62.5% | 50% | 1.31 | 29 | 0.606 | 0.24 | *-0.13* | *0.59* |
| | 37.5% | 2.66 | 29 | 0.080 | 0.48 | *0.16* | *0.82* |
| | 25% | 2.76 | 29 | 0.080 | 0.50 | *0.13* | *0.95* |
| | 12.5% | 4.23 | 29 | 0.003** | 0.77 | *0.46* | *1.17* |
| 50% | 37.5% | 2.21 | 29 | 0.176 | 0.40 | *0.05* | *0.72* |
| | 25% | 1.82 | 29 | 0.315 | 0.33 | *-0.01* | *0.80* |
| | 12.5% | 4.07 | 29 | 0.004** | 0.74 | *0.41* | *1.19* |
| 37.5% | 25% | 0.47 | 29 | 0.802 | 0.08 | *-0.46* | *-0.30* |
| | 12.5% | 2.76 | 29 | 0.080 | 0.50 | *0.19* | *0.84* |
| 25% | 12.5% | 3.21 | 29 | 0.029* | 0.59 | *0.31* | *0.91* |

**Supplemental Material - Table 2. Post-hoc comparisons between accuracies at different similarity levels averaged across younger and older adults (experiment 1).** Paired sample t tests were performed to compare between tactile patterns (t = test statistic, *df* = degrees of freedom, *p* (corr.) = Holm-Bonferroni-corrected *p*-value, *d* = Cohen's *d* and CI = confidence interval). Significance is indicated by *, ** or ***.

| | Mean ± SD | | | | 95% CI | |
|---|---|---|---|---|---|---|
| Completeness level | Younger | Older | $BF_{10}$ | error% | *Lower* | *Upper* |
| 100% | 6.67 ± 14.20 | 18.75 ± 25.77 | 2.17 | $1.143*10^{-5}$ | *0.05* | *1.09* |
| 87.5% | 10.83 ± 21.48 | 18.33 ± 32.29 | 0.646 | $7.193*10^{-7}$ | *0.02* | *0.82* |
| 75% | 2.92 ± 20.64 | 13.75 ± 30.98 | 1.051 | 0.001 | *0.03* | *0.94* |
| 62.5% | 4.58 ± 30.76 | 3.33 ± 28.02 | 0.282 | $3.255*10^{-4}$ | *0.01* | *0.61* |
| 50% | 0.00 ± 24.33 | 4.58 ± 23.33 | 0.504 | $2.968*10^{-5}$ | *0.01* | *0.76* |

**Supplemental Material - Table 3. Results of comparison of pattern completion bias between younger and older adults (experiment 2).** Bayesian independent-sample t tests were performed to compare the bias score at each completeness level between age groups ($BF_{10}$ = Bayes Factor, error% = error rate, CI = credible interval). The alternative hypothesis is older > younger on the bias score at each completeness level.
